# *Abelmoschus esculentus* subfractions ameliorate hepatic lipogenesis and lipid uptake via regulating dipeptidyl peptidase-4—With improving insulin resistance

**DOI:** 10.1371/journal.pone.0265444

**Published:** 2022-03-15

**Authors:** Chiung-Huei Peng, Yaw-Bee Ker, Hsin-Hua Li, Sing-Hua Tsou, Chih-Li Lin, Chien-Ning Huang

**Affiliations:** 1 Division of Basic Medical Science, Hungkuang University, Taichung, Taiwan; 2 Department of Food Science and Technology, Hungkuang University, Taichung, Taiwan; 3 General Education Center, National Taiwan University of Sport, Taichung, Taiwan; 4 Institute of Medicine, Chung-Shan Medical University, Taichung, Taiwan; 5 Department of Medical Research, Chung Shan Medical University Hospital, Taichung, Taiwan; 6 Department of Internal Medicine, Chung-Shan Medical University Hospital, Taichung, Taiwan; Auburn University, UNITED STATES

## Abstract

Nonalcoholic fatty liver disease (NAFLD) is recognized as the liver component of metabolic syndrome. The regulation of hepatic lipid should be emphasized to prevent accompanying illness. As AMP-activated protein kinase (AMPK) and sterol regulatory element binding protein (SREBP) regulate lipid metabolism, CD36 and fatty acid synthase (FAS) promote lipid uptake and lipogenesis respectively, while acetyl-CoA carboxylase (ACC) is an indicator of negative feedback. The increase of IRS-1 phosphorylation at the residue ser307 (p-ser307-IRS-1) and decrease of p-ser473-Akt (p-Akt) are viewed as the insulin resistance markers, and our previous reports suggested dipeptidyl peptidase-4 (DPP-4) mediates insulin resistance, the crucial factor of metabolic syndrome. *Abelmoschus esculentus* (AE) fruit is well-known for its antidiabetic utility. We had isolated several AE subfractions by successive steps, and found that F1 and F2 were especially valid in suppressing DPP-4 signaling. Since little is known if AE works on NAFLD, now we first attempt to investigate whether AE is useful to attenuate hepatic lipogenesis and lipid uptake in liver cells, along with improving the metabolic targets. We demonstrated that AE subfractions attenuated the hepatic lipid accumulation induced by free fatty acids. Treatment of AE alleviated FAS and returned the level of p-ser79-ACC (p-ACC). Although F1 was more effective on AMPK, F2 seemed more stable to attenuate SREBP-1. Moreover, as fatty acids stimulated the expression of CD36, F2 showed a superior effect to down-regulate the lipid uptake. Both AE subfractions reduced the generation of ROS, decreased the level of p-ser307-IRS-1, and restored the expression of p-Akt. Moreover, treatment of DPP-4 inhibitor linagliptin revealed that, AE could prevent the hepatic lipogenesis, oxidative burden, and the related insulin resistance via downregulating DPP-4. In conclusion, the present investigation revealed that AE, especially F2, is potential to be developed as adjuvant to prevent NAFLD.

## Introduction

Nonalcoholic fatty liver disease (NAFLD), which covers a wide spectrum of manifestations including liver steatosis, is recognized as the liver component of metabolic syndrome [1]. As a critical modulator, liver plays an essential role to regulate plasma lipid level, while the overload of hepatic lipid uptake burdens liver function and relates to the subsequent development of inflammation and fibrosis [2]. It was reported lipotoxicity and oxidative stress are the important mediators in NAFLD pathogenesis [3]. Hence the regulation of hepatic lipid metabolism should be emphasized to prevent accompanying illness.

Fatty acid synthase (FAS), the enzyme regulating triacylglyceride synthesis, is indicated as an important marker of lipogenesis, while fatty acyl CoA formed by long or short chain fatty acids, are negative feedback inhibitors of Acetyl-CoA carboxylase (ACC). ACC is the enzyme catalyzing the carboxylation of acetyl-CoA to produce malonyl-CoA, which is the important substrate of the initiation step of fatty acid synthesis [4], Apart from endogenous fatty acid synthesis, the uptake of fatty acid would be bound to lipid metabolism. CD36 is a wide-expressed transmembrane glycoprotein serves many functions, including the uptake of the long-chain fatty acid across cell membranes. The rate of uptake is primarily governed by the presence of CD36 at cell surface, regulated by the subcellular vesicular recycling from endosomes to the plasma membrane [5].

Sterol regulatory element binding protein (SREBP) is a transcription factor which regulates lipid metabolism and consists of isoforms. The polyunsaturated fatty acid was shown to inhibit maturation of SREBP-1, thus decreasing the downstream transcription of FAS [6]. Many previous reports displayed that SREBP was regulated by AMP-activated protein kinase (AMPK). The increase of AMPK phosphorylation prevented SREBP-1 expression in liver cells [7]. Accordingly, the anti-diabetic agent Metformin, also decreased hepatic SREBP-1 via regulating AMPK, and therefore lowered the hepatic lipid content [8].

Insulin resistance is one of the crucial factors of metabolic syndrome, which leads to the development of type 2 diabetes, thus results in severe medical problems in modern societies [9]. The overabundance of oxidative stress, directly correlating with increase of fat accumulation, is connected to the multifactorial etiology of insulin resistance [10]. The evidences show that there are tight associations between insulin resistance and hepatic steatosis, while the pathogenesis is not completely defined [9]. Insulin action involves a series of cascades. After binding to its receptor, insulin elicits tyrosine phosphorylation of insulin receptor substrates (IRS), leading to the activation of phosphatidylinositol 3-kinase/Akt (PI3K/Akt). However, the increase of IRS-1 phosphorylation at the residue ser307 (p-ser307-IRS-1) hinders the insulin response and glucose utilization, thus be viewed as an insulin resistance marker [4]. Recently, dipeptidyl peptidase-4 (DPP-4, a serine protease) inhibitors have emerged as a useful tool for treating type 2 diabetes. Our previous reports showed that DPP-4 activity is critical to mediate the downstream insulin resistance signals [11].

Abelmoschus esculentus (AE) fruit is well-known in folklore medicine for the antidiabetic utility, but the substantial mucilage makes it difficult to test the active components. In our previous reports, we isolated a few subfractions from AE using a series of successive extraction steps, and found that subfractions F1 (containing mainly quercetin glucosides and triterpene esters) and F2 (having large amounts of carbohydrates and polysaccharides) were especially valid in suppressing DPP-4 signaling [12]. F1 and F2 prevented cell apoptosis by attenuating DPP-4 and improving the insulin resistance [13,14]. In neurons, by regulating DPP-4 activity and insulin resistance signals, F1 and F2 reduced the production of Aβ and tau [15]. Furthermore, using the type 2 diabetic rat, we reported the subfractions of AE could take advantage at different targets respectively [16,17]. However, little is known if AE prevent liver steatosis along with improving these metabolic targets.

In the present study, using an *in vitro* model co-induced with saturated and unsaturated fatty acids, we first investigated whether AE is useful to alleviate DPP-4 cascades, and regulates the lipogenesis and lipid uptake in liver cells.

## Results

### Concentrations of FFAs applied were tested for the safety range

**Fig 1:**
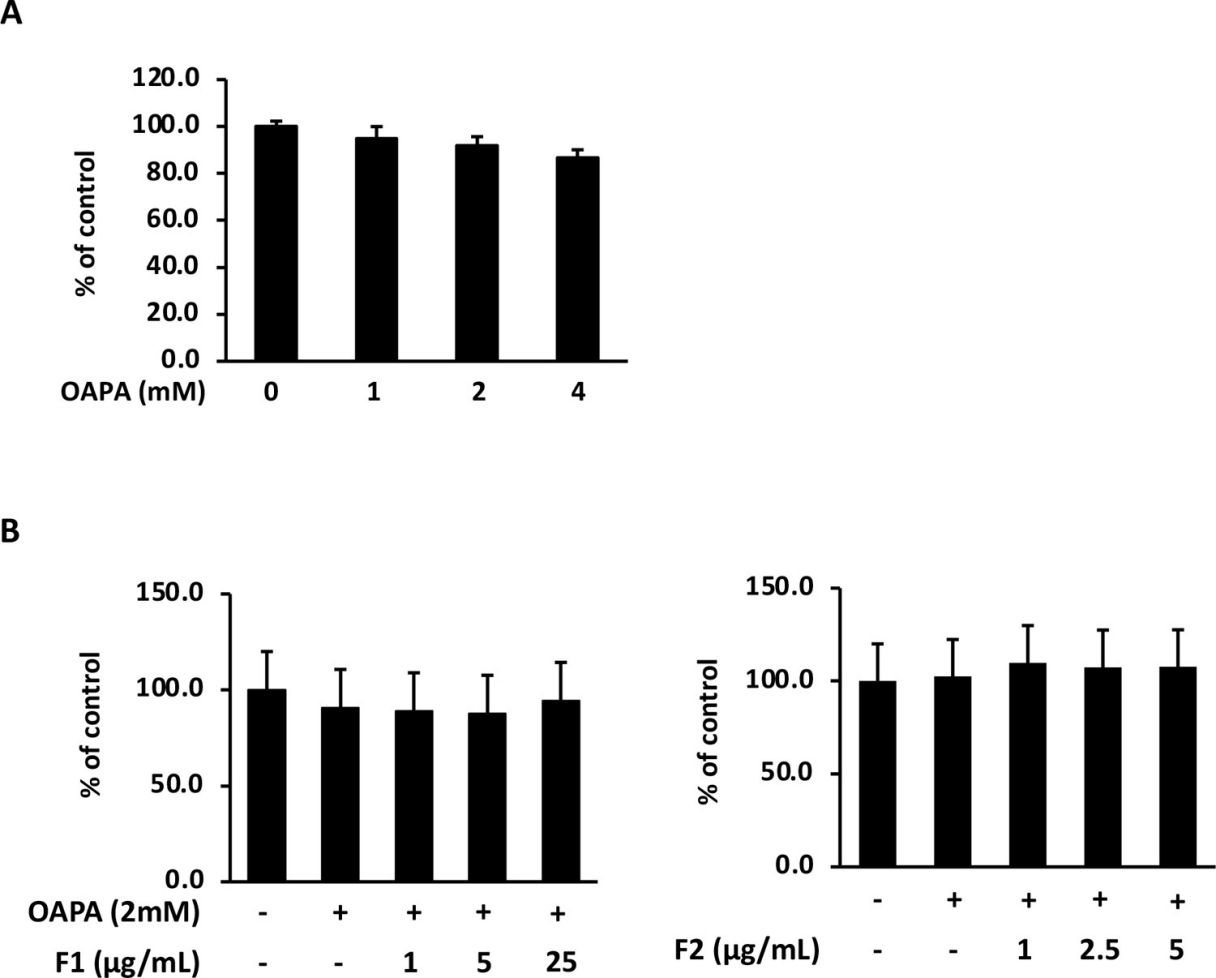
Cytotoxicity of OAPA and AE subfractions. (A) HepG2 cells were incubated for 24 h with or without various concentrations of OAPA. The cell viability was analyzed with MTT and calculated as a percentage compared with which of the control group. (B) After that, cells were treated with 2 mM of OAPA added with different concentrations of F1 or F2. All the data were presented as means ± SD (n = 3), and statistically analyzed with ANOVA. No statistical difference was found.

In the present study, OAPA (oleic acid and palmitic acid with the ratio of 2:1) was applied to induce insulin resistance but not cell death. At beginning of the experiment, MTT was used to test the operating dose of OAPA. Fig 1A showed that OAPA did not reduce the cell viability of HepG2 even at the dose of 4 mM. As well, co-treated with 2 mM of OAPA, F1 and F2 displayed no cytotoxicity to HepG2 cells at the dose of 25 μg/mL and 5 μg/mL, respectively (Fig 1B). Hence, the above dose ranges were chosen and applied in the following manipulation.

### AE subfractions attenuate hepatic lipid accumulation induced by FFAs

**Fig 2:**
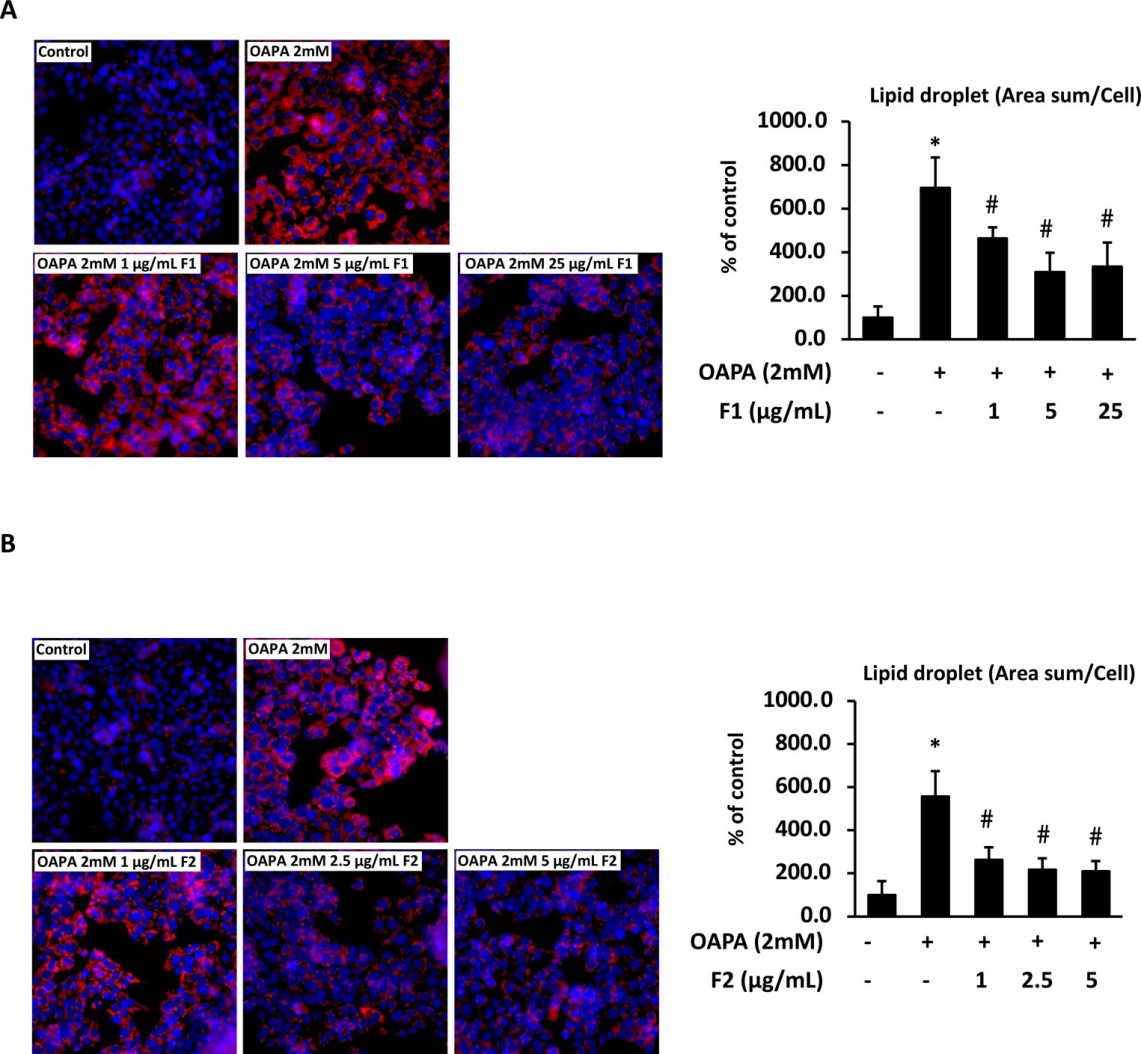
Effect of AE on attenuating hepatic lipid accumulation. HepG2 cells were incubated for 24 h with or without 2 mM of OAPA, and with different concentrations of F1 (A) and F2 (B). Cells were stained with Nile red (lipid droplets, red) and Hoechst (nucleus, blue), and observed with immunofluorescence microscopy (100x magnification). Data were presented as means ± SD (n = 3), and analyzed with ANOVA. *p < 0.05, compared with the control. #p < 0.05, compared with the OAPA-treated only.

The immunofluorescence image showed that OAPA induced the lipid accumulation. Fig 2 showed that OAPA increased the lipid droplets approximately 6 folds. Conversely, treatment of all the applied doses of F1 (1, 5 and 25 μg/mL) and F2 (1, 2.5 and 5 μg/mL) significantly decreased the lipid droplets, while F2 seemed to be more superior and stable, decreasing the lipid droplets dose-dependently. These findings implied that AE subfractions could affect the lipid metabolism, maybe including the endogenous lipogenesis or the lipid uptake.

### AE subfractions attenuate endogenous lipogenesis and lipid uptake

**Fig 3:**
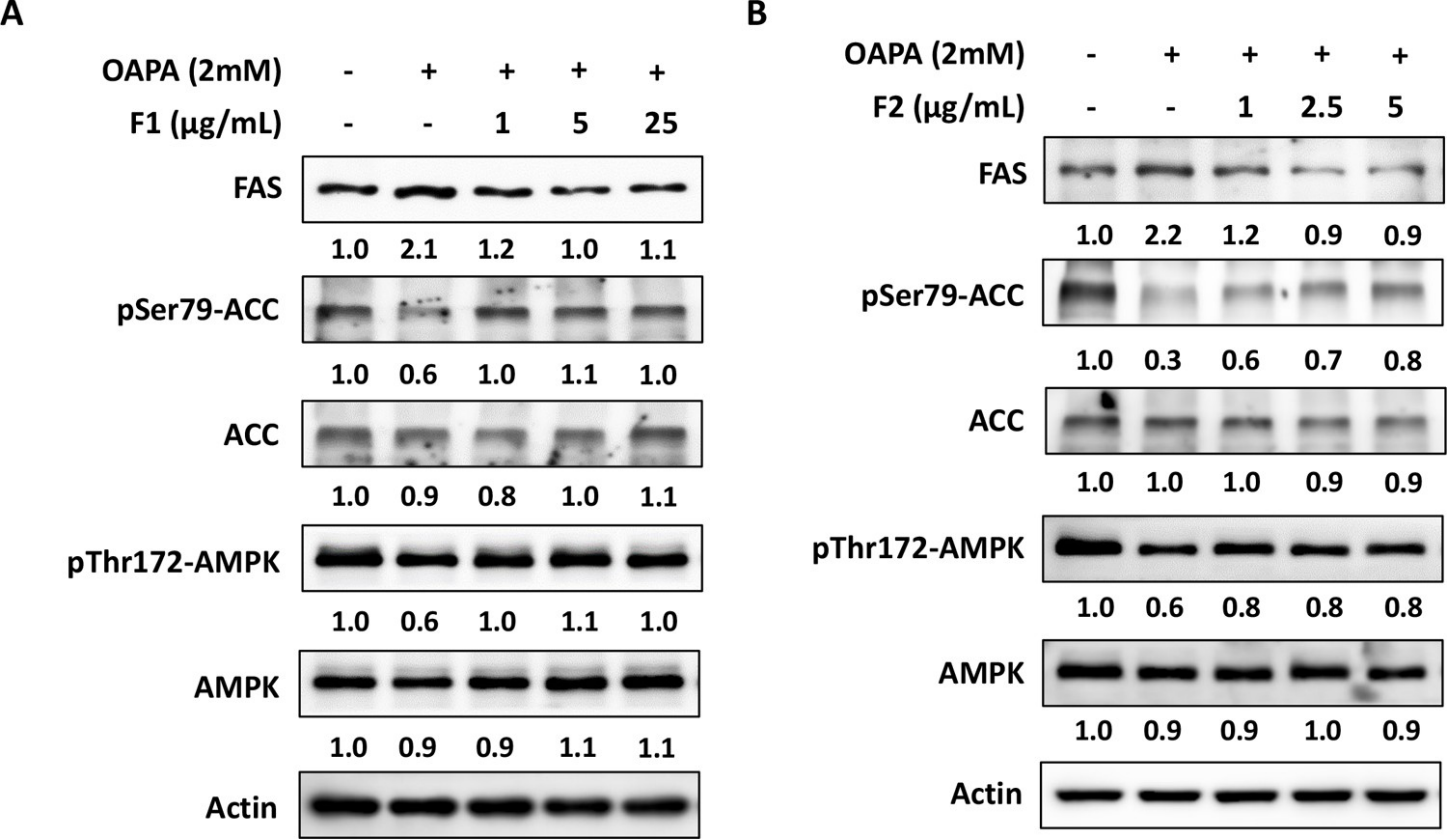
Effect of AE on attenuating lipogenesis. HepG2 cells were incubated for 24 h with or without OAPA, and with different concentrations of F1 (A) and F2 (B). The protein levels of FAS, p-ACC, ACC, p-AMPK and AMPK were analyzed by western blotting and quantified by densitometer. Three independent experiments were conducted which showed the similar pattern of changes.

**Fig 4:**
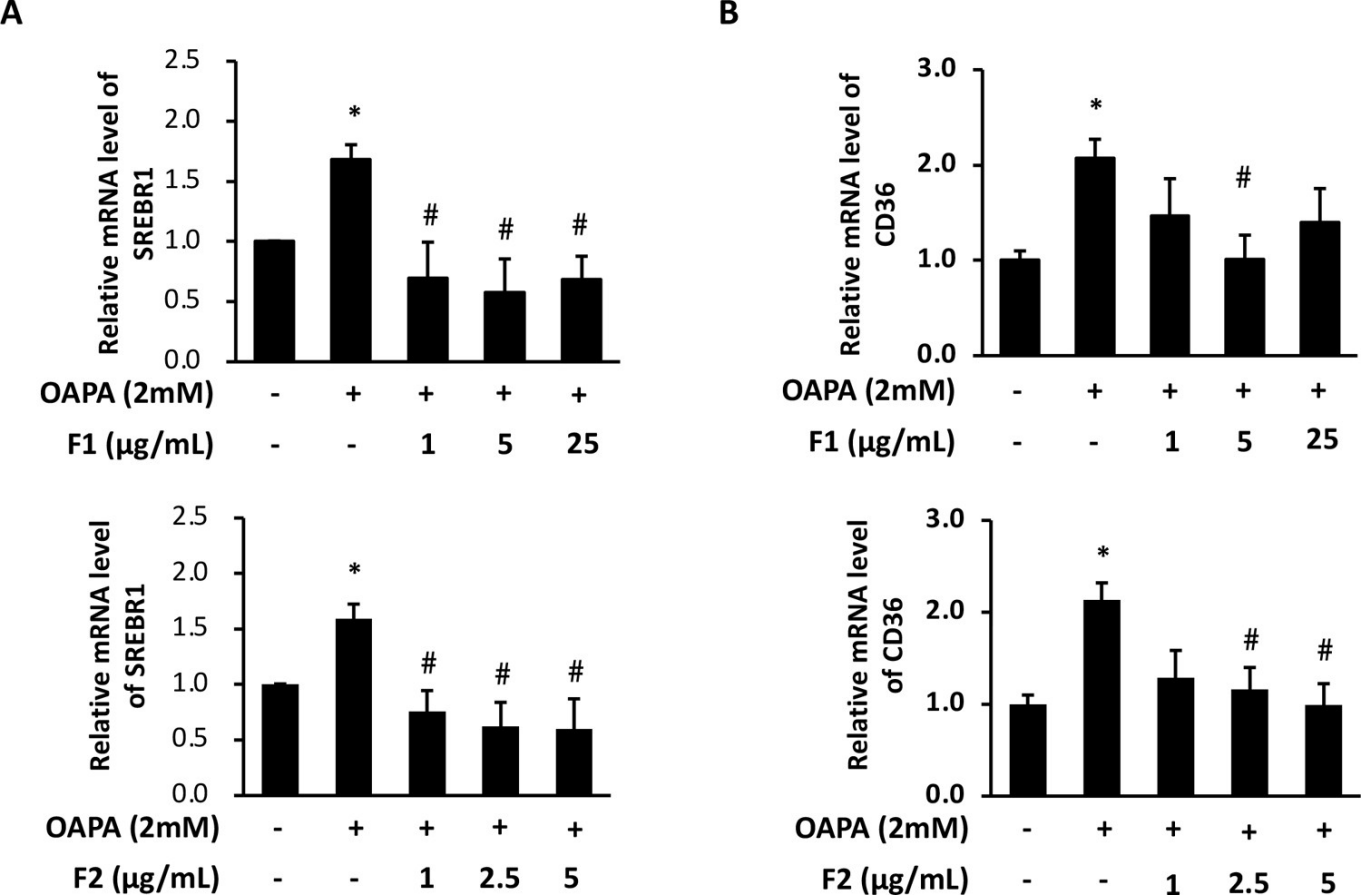
Effect of AE on regulating SREBP-1 and attenuating lipid uptake. HepG2 cells were incubated for 24 h with or without OAPA, and with different concentrations of F1 and F2. SREBP-1 (A) and CD36 (B) mRNA levels were analyzed with RT-PCR. Data were presented as means ± SD (n = 3), and analyzed with ANOVA. *p < 0.05, compared with the control. #p < 0.05, compared with the OAPA-treated only.

In Fig 3, OAPA increased the protein level of FAS more than 2 folds, while decreased p-ACC. Treatment of F1 alleviated the expression of FAS, and reversed the level of p-ACC. F2 displayed the similar effect, particularly at 5 μg/mL. The process of lipogenesis is upstream-regulated by AMPK and SREBP-1, hence these two markers were also evaluated. Figs 3 and 4A showed that OAPA decreased the activation of AMPK, and increased the expression of SREBP-1. Treatment of F1 or F2 significantly restored the level of p-thr172-AMPK (p-AMPK) and SREBP-1, while F1 showed more useful to upregulate p-AMPK, and F2 seemed slightly superior to downregulate SREBP-1.

Fig 4B showed that OAPA increased the level of CD36 to more than 2 folds. Although both F1 and F2 alleviated CD36, F2 seemed more stable, and displayed a superior effect to downregulate the marker of fatty acid uptake.

### AE subfractions attenuate the generation of oxidative stress

**Fig 5:**
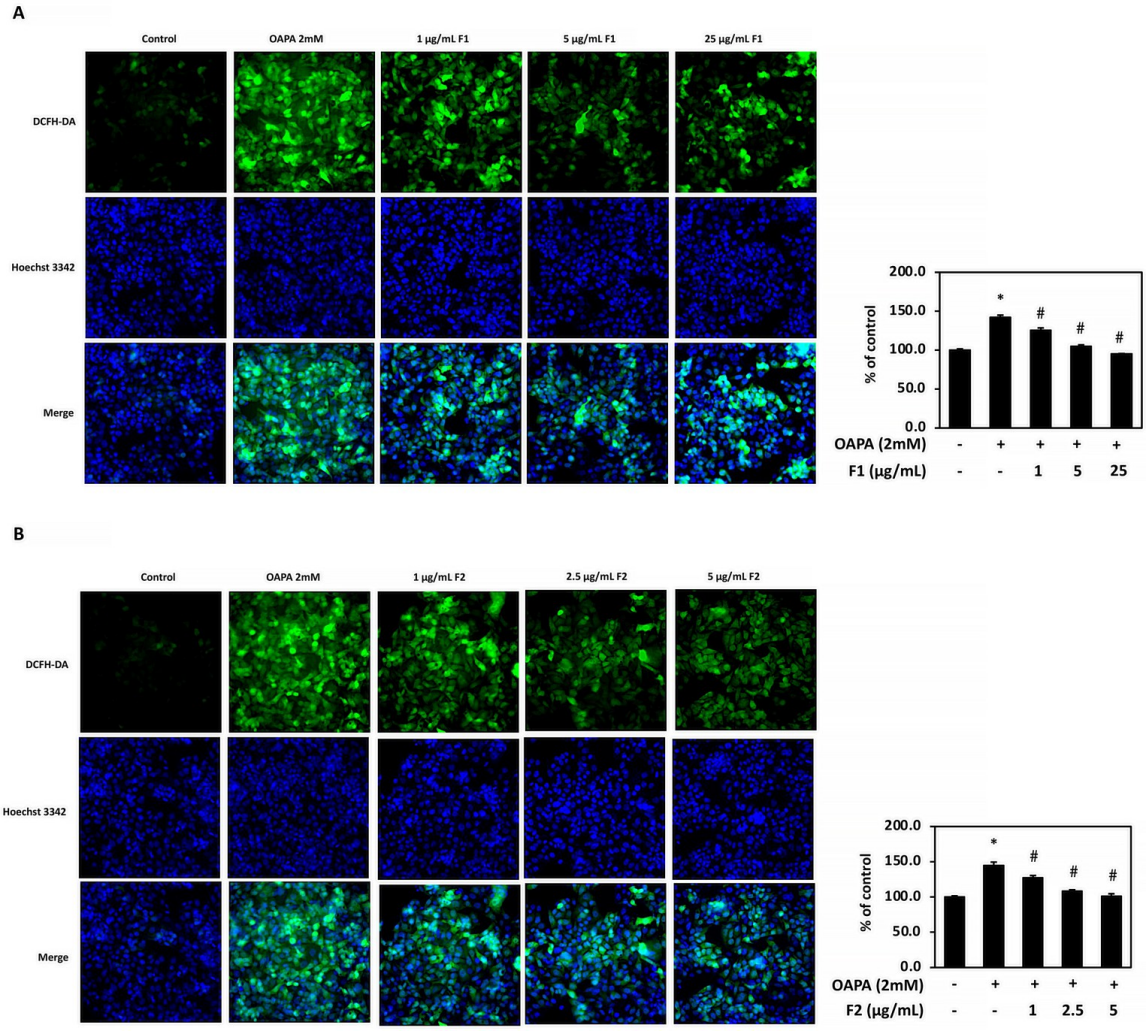
Effect of AE on attenuating oxidative stress. HepG2 cells were incubated for 24 h with or without 2 mM of OAPA, and with different concentrations of F1 (A) and F2 (B). Cells were stained with DCFH-DA (ROS, green) and Hoechst (nucleus, blue), and analyzed with Immunofluorescence (200x magnification). Data were presented as means ± SD (n = 3), and analyzed with ANOVA. *p < 0.05, compared with the control. #p < 0.05, compared with the OAPA-treated only.

The immunofluorescence image showed that OAPA induced the generation of ROS. Fig 5 demonstrated that OAPA increased the lipid droplets approximately 1.5 folds, while treatment of all the applied doses of F1 (1, 5 and 25 μg/mL) and F2 (1, 2.5 and 5 μg/mL) significantly decreased the oxidative stress.

### AE subfractions attenuate hepatic expression of DPP-4

**Fig 6:**
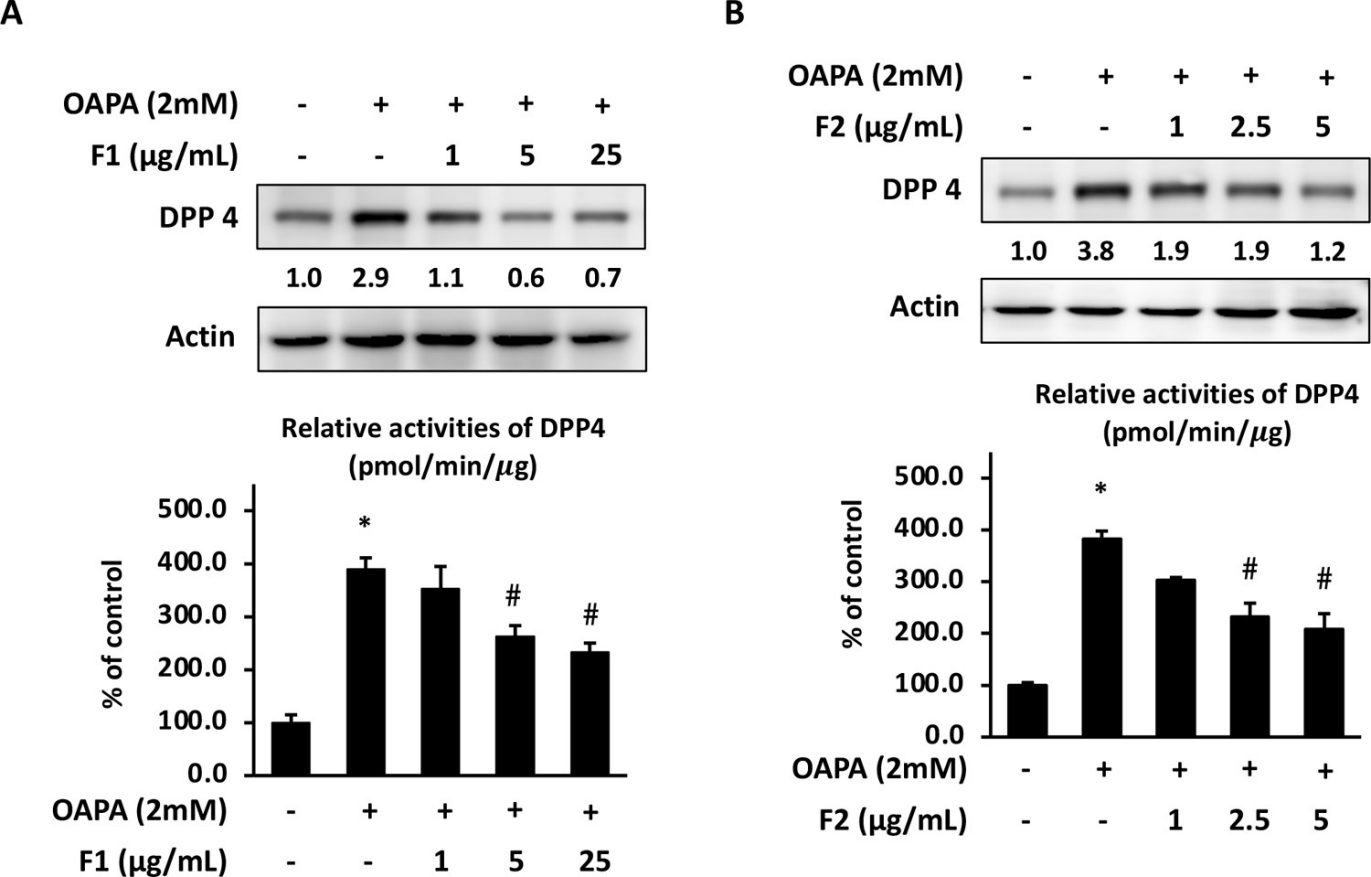
Effect of AE on attenuating hepatic DPP-4. HepG2 cells were incubated for 24 h with or without various concentrations of OAPA. The protein level and activity of DPP-4 were analyzed. For the DPP-4 activity, data were presented as means ± SD (n = 3), and analyzed with ANOVA. *p < 0.05, compared with the control. #p < 0.05, compared with the OAPA-treated only. For the western blotting, three independent experiments were conducted which showed the similar pattern of changes.

Fig 6 showed that OAPA increased the expression of DPP-4. At the dose of 2 mM, OAPA had already enhanced the protein level and activity of DPP-4 approximately 3 folds. It was shown that treatment with either F1 or F2, decreased the expression of DPP-4. At 25 μg/mL, F1 decreased 40% of DPP-4 activity; at 5 μg/mL, F2 almost decreased 50% of DPP-4 activity.

### AE subfractions attenuate hepatic insulin resistance signals

**Fig 7:**
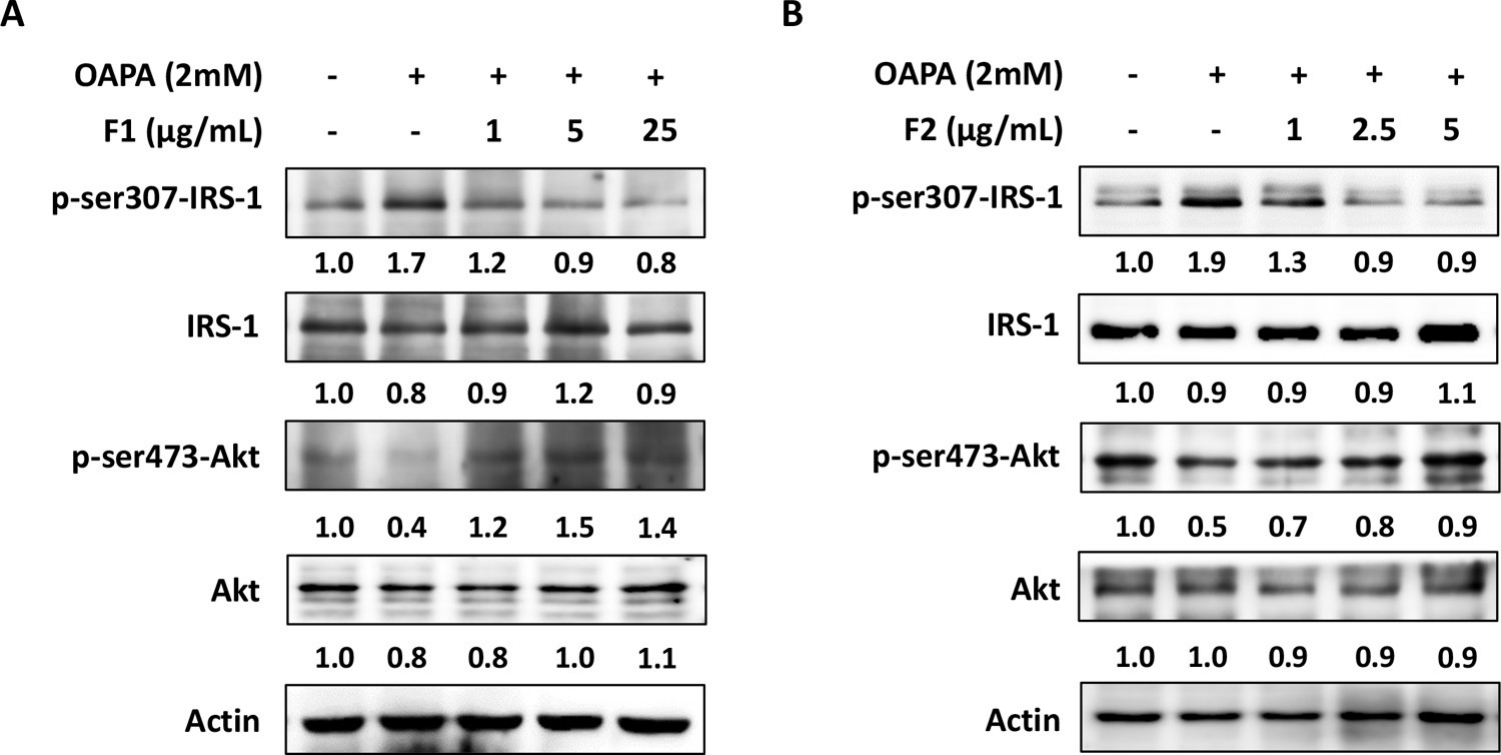
Effect of AE on attenuating hepatic insulin resistance signaling. HepG2 cells were incubated for 24 h with or without various concentrations of OAPA. The expressions of p-ser307-IRS-1, IRS-1, p-AKT and AKT were analyzed by western blotting and quantified by densitometer. Three independent experiments were conducted for the blotting, which showed the similar pattern of changes.

Likewise, OAPA increased the phosphorylation of IRS-1. At both 2 mM and 4 mM, OAPA elevated p-ser307-IRS-1 about 1.6 folds and 1.8 folds, respectively, and significantly reduced the expression of p-Akt. Both F1 and F2 decreased p-ser307-IRS-1 but increased p-Akt dose-dependently. 25 μg/mL of F1 and 5 μg/mL of F2 decreased p-ser307-IRS-1 more than 50%, and increased p-Akt more than 2 folds (Fig 7).

These data of Figs 6 and 7 revealed that 2 mM of OAPA could trigger the hepatic insulin resistance signals by enhancing DPP4 and p-ser307-IRS-1, and reducing p-Akt. The AE subfractions F1 and F2 effectively attenuated DPP4 activation and the cascades of insulin resistance in liver cells.

### DPP-4 is pivotal to mediate hepatic lipid metabolism, oxidative stress, and insulin resistance

**Fig 8:**
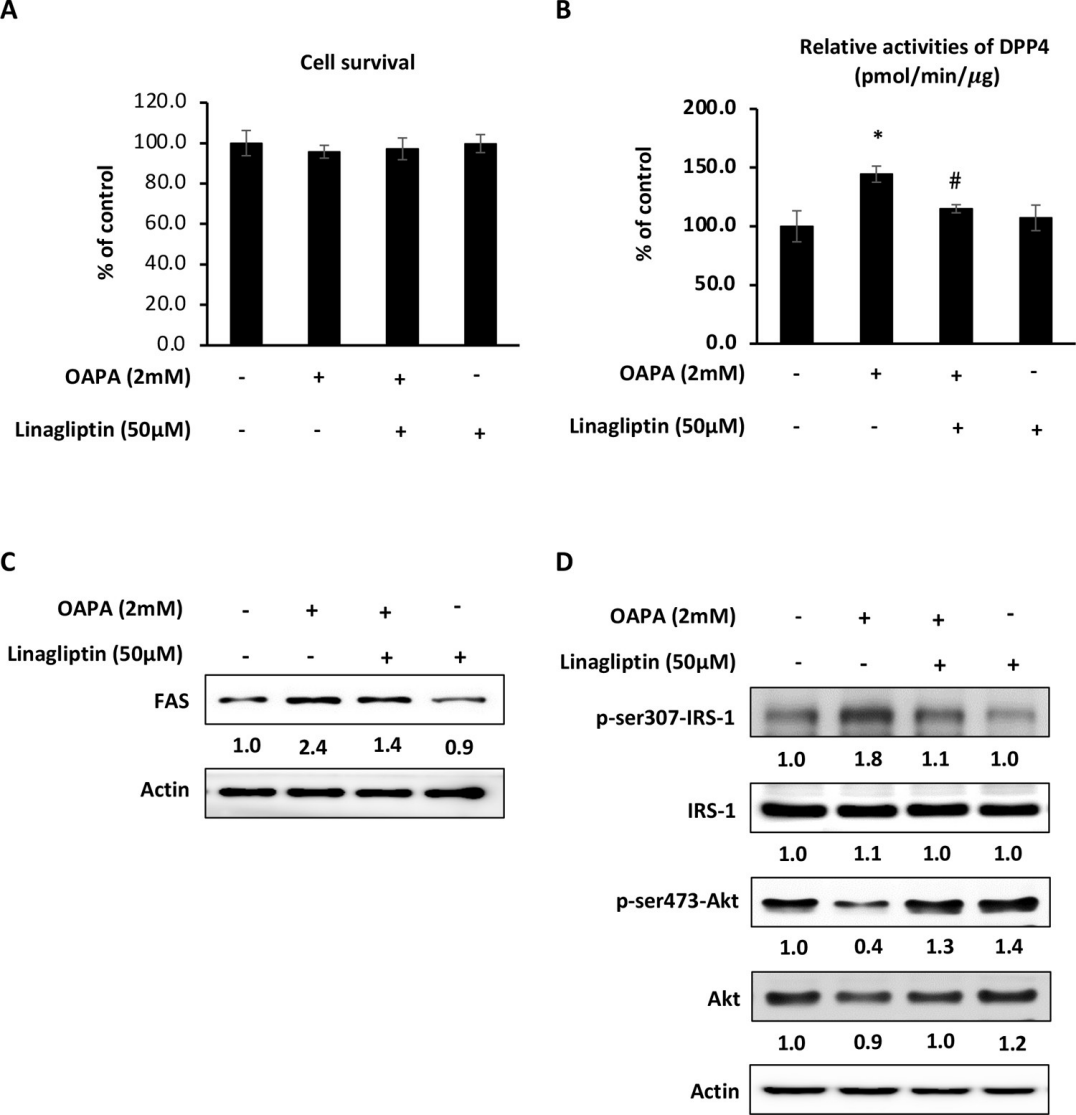
DPP-4 mediating insulin resistance and lipogenesis. HepG2 cells were incubated with OAPA and 50 μM of linagliptin for 24 h, and then analyzed the cytotoxicity (A), DPP-4 activity (B), the expression of FAS (C) and insulin resistance (D) signals. For the cytotoxicity and DPP-4 activity, data were presented as means ± SD (n = 3), and analyzed with ANOVA. *p < 0.05, compared with the control. #p < 0.05, compared with the OAPA-treated only. For the immunoblotting, three independent experiments were conducted which showed the similar pattern of changes.

The DPP-4 inhibitor linagliptin was added to clarify whether DPP-4 is associated with the hepatic lipid metabolism. Fig 8 showed that 50 μM of linagliptin displayed no cytotoxicity to HepG2 cells, and effectively reduced the activity of DPP-4. Accompanying with the inhibition of DPP-4, the lipogenesis marker FAS and insulin resistance signals were apparently suppressed.

## Discussion

In the present study, we demonstrate AE attenuates the hepatic lipid accumulation induced by free fatty acids. Treatment of AE alleviates FAS and returns the level of p-ACC. Although F1 is more effective on AMPK, F2 seems more stable to attenuate SREBP-1. Moreover, as fatty acids stimulate the expression of CD36, F2 demonstrates a superior effect to down-regulate the lipid uptake. Both AE subfractions reduce the generation of ROS, decrease the level of p-ser307-IRS-1, and recover the expression of p-Akt. By ameliorating DPP-4, AE could prevent the hepatic lipogenesis, oxidative burden, and the related insulin resistance.

NAFLD is now the most frequent chronic liver disease, affecting more than 25% of the general population [18]. There is accumulating evidence supports the association between NAFLD and the metabolic syndrome. Most of the patients with NAFLD have higher ratio of metabolic syndrome [19]. On the other hand, greater number of metabolism syndrome components was significantly associated with higher risk of NAFLD [20]. It was shown that the association was stronger in non-obese individuals, especially in women [21].

Insulin resistance is recognized as a key to link both NAFLD and metabolic syndrome. Insulin resistance is associated with increased circulating free fatty acids and excessive fat accumulation in ectopic tissues, such as liver, and promotes inflammation and endoplasmic reticulum stress. This in turn aggravates the insulin resistant state, thus constituting a vicious cycle [22]. Smith *et al*. reported the increase of circulating glucose and insulin stimulate hepatic lipogenesis in individuals with NAFLD [23]. Liu *et al*. reported the reduction of intracellular lipid content in liver and skeletal muscle suppressed inflammation markers, hence alleviating the insulin resistance in high fat diet-induced obese mice [24]. In fact, the oxidative stress and release of pro-inflammatory cytokines are major consequences of the hepatic lipid overload [25]. It was reported direct exposure to the oxidant stress led to diminished insulin signaling and inactive glucose transport, especially in the mammalian skeletal muscle [26]. In the present *in vitro* study, we displayed the insulin resistance is consistently with the hepatic lipid uptake and lipogenesis. Although whether the insulin resistance signals regulate the lipid metabolism or the converse cannot be defined, our data revealed that DPP-4 plays a critical role and mediates both the insulin resistance and hepatic lipid accumulation.

As a useful prescription for type 2 diabetes, the action of DPP-4 is based on inhibiting the degradation of type 1 glucagon-like peptide (GLP-1), an incretin stimulates the glucose-dependent insulin secretion of β cells. Besides their action on the pancreas, GLP-1 and DPP-4 have direct effects on various organs including the heart, blood vessel, and kidney [27]. It is worth noting that DPP-4 affects insulin resistance cascades, independent of the degradation of GLP-1. Furthermore, there exist strong associations between DPP-4 and NAFLD. In the mice fed with monosodium glutamate (MSG) and a high-fat diet (HFD), treatment of DPP-4 inhibitor attenuated hepatic lipogenesis by activating AMPK and downregulating the genes involved in lipogenesis [28]. In NAFLD patients, the hepatic DPP4 mRNA expression was significantly greater than that in the control subjects [29]. Our data found that DPP-4 regulates FAS, the marker of lipogenesis. Hence AE inhibited DPP-4, and the downstream lipogenesis.

A recent report suggested AE decreased serum and hepatic lipid in a mouse model, and the hypolipidemic activity of AE was mediated by reducing lipogenesis through SREBP1c and FAS [30]. As we previously reported, AE subfraction F1 and F2 are mainly composed of quercetin glycosides and polysaccharides, respectively. It was found that treatment of quercetin attenuated the fatty acid uptake, trafficking-related gene expression, and lipid peroxidation in nonalcoholic steatohepatitis of MCD-induced mice [31]. The extract rich in quercetin glucoside improved metabolic syndrome and possessed liver-protective effect by reducing the inflammatory cytokines and oxidative stress [32]. Actually, we have also tested the sub-subfractions of F1. The results showed that the effect of F1, could be partly attributed to the portion rich in quercetin glycosides, which showed a good effect to reduce the hepatic lipid (data not shown). As well, the polysaccharides could also be liver-protective. It was demonstrated that the polysaccharide of *Radix Hedysari* ameliorated hepatic lipid content and improved liver damage by activating AMPK and downregulating SREBP-1c, thus reduced lipogenesis while increased lipolysis [33]. Our data revealed that at small effective dose, F2 took advantage especially on the regulation of CD36 and SREBP-1, and this could be attributed to the composed polysaccharides.

As we know, this is the first time AE was reported useful to improve the fatty liver associated with insulin resistance, via regulating DPP-4. Despite both subfractions modulate the hallmarks involved in lipid metabolism, F2 seems more feasible to be developed. In conclusion, we demonstrated that AE would be a potential adjuvant to prevent NAFLD.

## Materials and methods

### Chemicals

We purchased reagents, such as palmitic acid (PA), oleic acid (OA), Hoechst 33342, Nile red, and 2’,7’-dichlorodihydrofluorescein diacetate (H2-DCFDA), from Sigma Aldrich (München, Germany). Pure linagliptin was provided by Boehringer Ingelheim Pharmaceuticals (Biberach, Germany). All the chemicals were prepared by dissolving phosphate buffer saline solutions stored at -20°C until needed for use in experiments.

### Preparation of the subfractions of AE

AE fruits were purchased from Chiayi, Taiwan, and the subfractions F1 and F2 were prepared according to a succession of procedures as we reported [12]. F1, extracted by 95% alcohol and analyzed with LC-MS/MS, contains at least 10 compounds including quercetin glucosides (4.901mg/g DW) and pentacyclic triterpene ester (4.301mg/g DW). F2, extracted by water at 90°C, has large amount of polysaccharides. Analyzed with gel filtration chromatography (GPC), the mean molecular weight of F2 was estimated as 671 kDa, rich in uronic acid (23.14%), galactose (18.92%), glucose (18.26%) and myoinositol (14.21%); rhamnose, fucose and glucosamine were also found [12].

### Cell culture

The cell line HepG2 was obtained from American Type Culture Collection, and cultured in Dulbecco’s minimal essential medium (DMEM) supplemented with 10% of fetal bovine serum, 100 unit/mL penicillin, 100 μg/mL streptomycin, and 2 mM glutamine (HyClone, Thermo Scientific) at 37°C, in a humidified atmosphere of 95% air-5% CO_2_. To induce FFAs-overloading, cells at 70% confluency were washed with PBS, and then were exposed to a long-chain mixture of FFAs (mixture of oleic acid and palmitic acid, OAPA; with the ratio of 2:1). 10 mM FFAs stock solution was prepared in 0.1 M NaOH by heating to 70°C in a shaking water bath. Next, a 10% FFA-free bovine serum BSA (Sigma Aldrich) solution was prepared in H_2_O, and vortex mixed with FFAs stock solution for 10 sec followed by a further 10 min incubation at 55°C. The FFAs-BSA conjugate stock solution was cooled to room temperature and sterile filtered through a 0.45 μm pore membrane filter. It can be stable stored at -20°C for 3–4 weeks, where the stock solutions were heated for 15 min at 55°C, then cooled to room temperature before use.

### MTT assay

Cells were seeded at a density of 2 × 10^5^ cells/well on a 24 well-plate. After attachment, cells were incubated with OA/PA with or without different concentrations of F1 or F2 for 24 h. The medium was then changed, and cells were incubated with 0.5 mg/mL of 3-(4, 5-dimethyl-2-thiazol)-2, 5-diphenyltetrazolium bromide (MTT, CAS 298–93–1) for 2 h. The number of viable cells was proportional to the generation of formazan. Dissolved with isopropanol (1 mL/well) and centrifuged at 12000 rpm, each sample was added to a 96 well-plate (200 μL per well), and the absorbance was measured by the spectrophotometer at 563 nm (Hitachi, U-3210).

### Western blotting

Cells were harvested with the lysis buffer containing 50mM Tris HCl at pH 6.8, 10% of glycerol, 2% of SDS, and 5% of mercaptoethanol, and lysed by sonication afterwards. The lysates were centrifuged at 9300 g for 20 min at 4°C, and the supernatant was collected as protein samples. Quantified by Bradford assay, equal amount (50 μg) of each protein sample was subjected to 10% of SDS polyacrylamide gel electrophoresis, and transferred to the nitrocellulose membrane (Millipore, Bedford, MA, USA). The membranes were blocked in a 2% bovine serum albumin-based blocking buffer in TBS with 0.1% Tween20 for 2 h, then incubated with the primary antibody overnight at 4°C for the following targets: p-ser307-IRS-1, IRS-1, p-ser473-Akt, Akt, p-ser79-ACC, ACC, p-thr172-AMPK, AMPK, FAS and DPP-4. The antibody of DPP-4 was from Abcam (Cambridge, U.K.). Antibodies of IRS-1, Akt, pAkt, and ACC were from Santa Cruz (Santa Cruz, CA, USA). Antibody of pACC (07–303) was obtained from Sigma‐Aldrich (München, Germany). Antibodies of p-ser307-IRS-1, p-AMPK and AMPK were purchased from Cell Signaling Technology (Beverly, MA), and FAS and anti-FAS were from Upstate Biotechnology (Lake Placid, NY). After that, the membranes were washed three times with 0.1% Tween-20 in TBS; then incubated with the secondary antibody conjugated to horseradish peroxidase (GE Healthcare, Little Chalfont, Buckinghamshire, UK). Band detection was fulfilled by enhanced chemiluminescence with ECL detection reagents, and FUJFILM Las-3000 (Tokyo, Japan). Protein quantity was determined by densitometry with FUJFILM-Multi Gauge V2.2 software.

### DPP-4 activity assay

Cells were seeded at a density of 1 × 10^6^ on a 6 cm-dish. After treated with various conditions, cells of each well were treated with 100 μL of lysis buffer (containing 10mM HEPES at pH 7.5, 142.5mM KCl, 5mM MgCl_2_, 1mM EGTA, and 0.2% of NP-40), and centrifuged at 9300 g for 20 min at 4°C. The supernatants were then collected, and their protein concentrations were determined with Bradford assay. DPP4 activity of all the collected samples was measured with DPP4/CD26 assay kit (Enzo Life Sciences). Briefly, the chromogenic substrate of DPP-4 was hydrolyzed into dipeptide Gly-Pro and 4-nitroaniline, whose production was measured spectrophotometrically at 405 nm. The activity was normalized with the protein concentration, and leveled in proportion with the control. For analyzing the putative role of DPP-4, the clinical regimen linagliptin, was used to inhibit DPP-4 in the experiment.

### Assessments of lipid droplets

For evaluation of the intracellular lipid droplets, cells were stained with Nile red (1 μg/mL), and the nuclei were stained with Hoechst 33342. After 30 min-incubation, the staining medium was discarded, and cells were then washed twice with PBS. The intracellular lipid distribution was observed by fluorescence microscopy. Cell imaging was performed by high-content imaging and analysis solutions (ImageXpress® Micro Confocal system; Molecular devices).

### Reactive Oxygen Species (ROS) assay

To evaluate the generation of intracellular ROS, cells were seeded on glass coverslips and incubated with 10 μM of 2’, 7’-dichlorodihydrofluorescin diacetate (DCFH-DA) for 30 min at 37°C under 5% CO_2_. After incubation, the staining medium was discarded and cells were washed twice immediately with PBS. The intensity of fluorescence was imaged by a fluorescence microscopy (DP72/CKX41; Olympus), with the excitation wavelength 488 nm and emission wavelength 525 nm.

### Reverse Transcription-Polymerase Chain Reaction (RT-PCR)

To determine the expression of SREBP-1c and CD36 at transcription level, total RNA was extracted using RNeasy Kit (Qiagen, Germantown, MD, USA) and quantified spectrophotometrically. The cDNA was reverse transcribed with TProfessional Thermocycler Biometra (Göttingen, Germany); then the PCR amplification were performed by Power SYBR Green PCR Master Mix (Applied Biosystems) in accordance with the manufacturer’s instructions. The SREBP-1c primers were forward, 5’-AGGATGCTCAGTGGCACTG-3’, and reverse, 5’-GGATTGCACTTTCGAAGACGTG-3’, which amplified a 110 bp fragment, run for 31 cycles at 95°C for 1 min, 58°C for 1 min and 72°C for 1 min. The CD36 primers were forward, 5’- AGTGATGATGAACAGCAGCAACA -3’, and reverse, 5’- TGGGTTACATTTTCCTTGGCTAGA -3’, which amplified a 90 bp fragment, run for 40 cycles at 95°C for 1 min, 60°C for 1 min and 72°C for 1 min. The relative values of mRNA expression were obtained by Sequence Detection Systems software (Sequence Detection Systems 1.2.3‐7300 Real Time PCR System, Applied Biosystems) and standardized in comparison with those obtained for the relative expression of GAPDH.

### Statistical analysis

All data are presented as means ± standard error of the mean. The software SPSS v.12.0 was used to analyze the data. One-way ANOVA was performed (p < 0.05), and Bonferroni’s multiple comparison was used for post hoc test.

## Supporting information

S1 Raw images (PDF)
