# Supplementary figures and images for: Abelmoschus esculentus subfractions ameliorate hepatic lipogenesis and lipid uptake via regulating dipeptidyl peptidase-4—With improving insulin resistance
Source: PLoS One. 2022 Mar 15;17(3):e0265444. doi: 10.1371/journal.pone.0265444 (PMC8923473; doi:10.1371/journal.pone.0265444)

Fig. 3

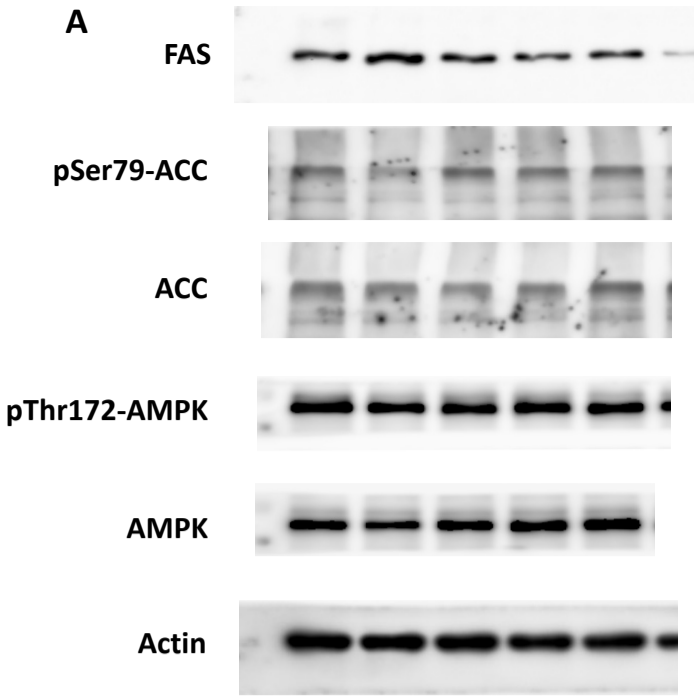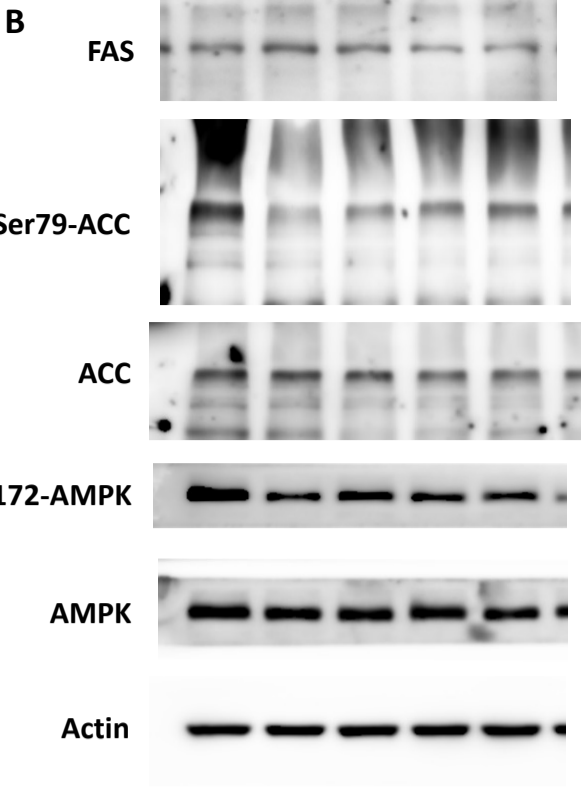

Fig. 6

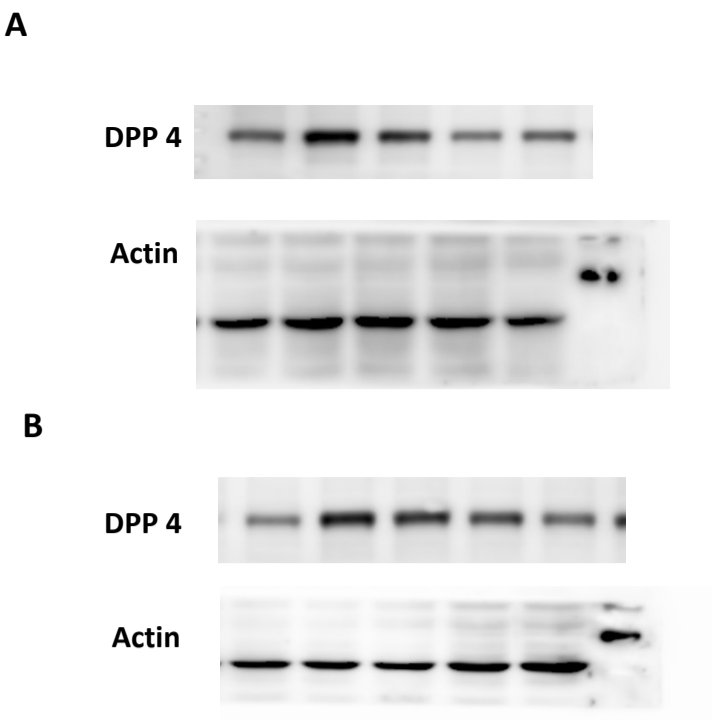

Fig. 7

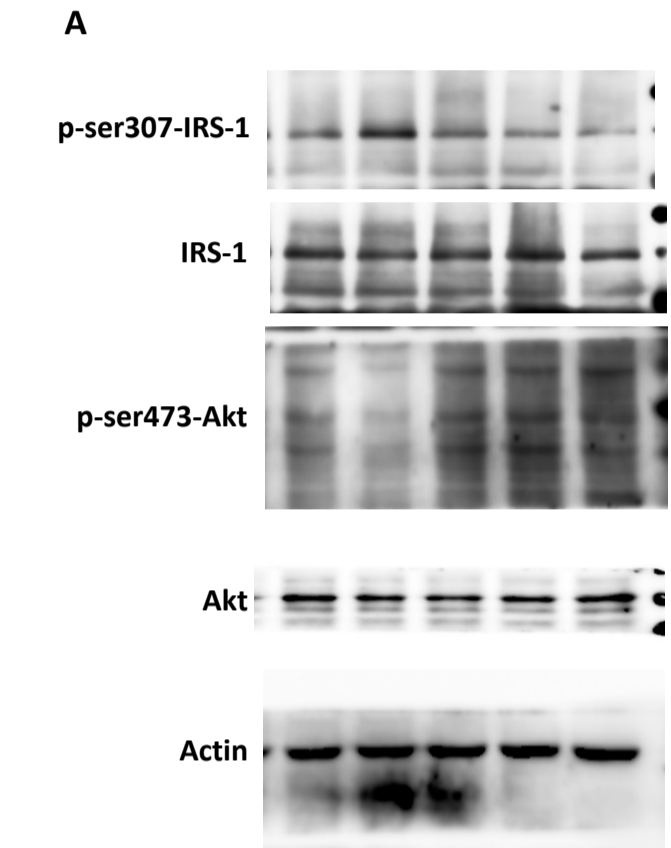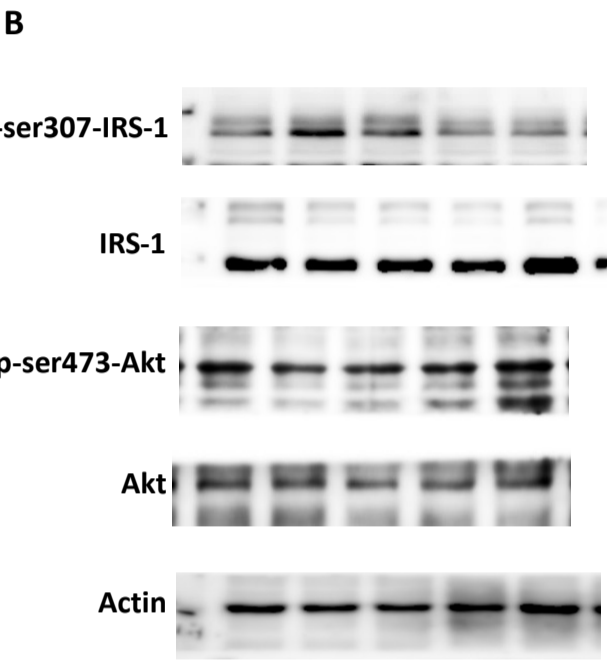

Fig. 8

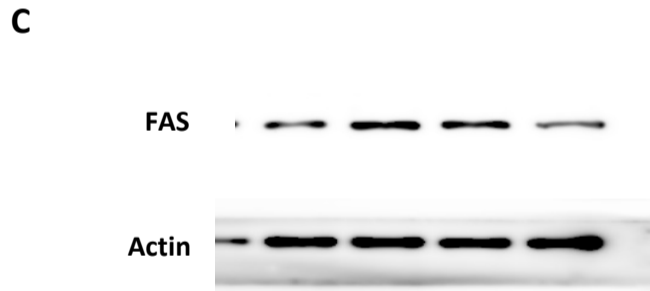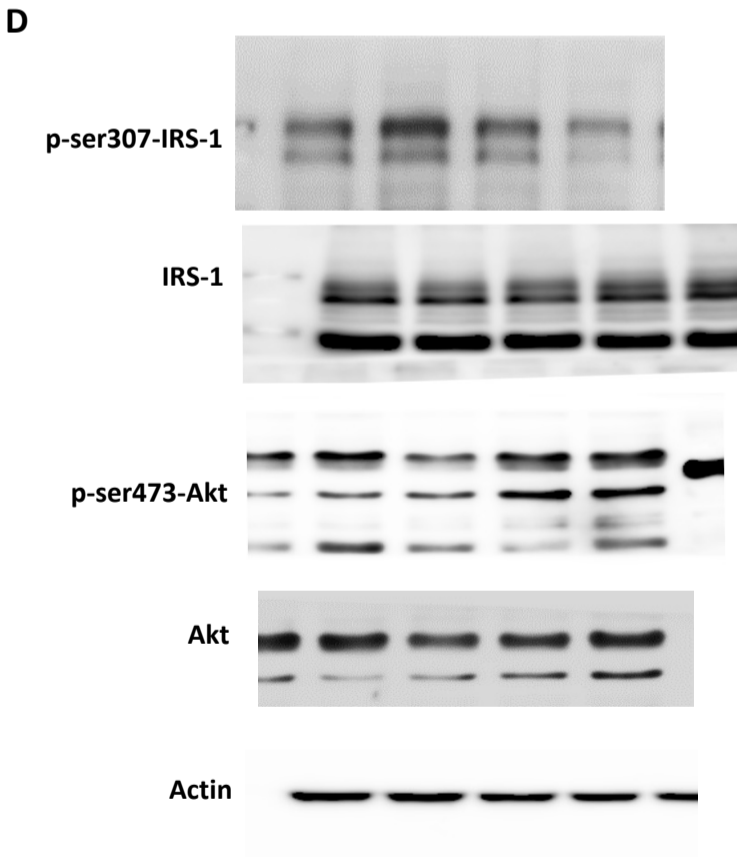

Supplement: S1 Raw images — (PDF) [file pone.0265444.s001.pdf]
